# Supplementary material for: Direct observation of imploded core heating via fast electrons with super-penetration scheme
Source: Nat Commun. 2019 Dec 9;10:5614. doi: 10.1038/s41467-019-13574-8 (PMC6901506; doi:10.1038/s41467-019-13574-8)
Supplement: Supplementary file 1 — Supplementary Information [file 41467_2019_13574_MOESM1_ESM.pdf]

Supplementary Information for

Direct observation of imploded core heating via fast electrons with super-penetration scheme

Gong et al.

Supplementary Figures

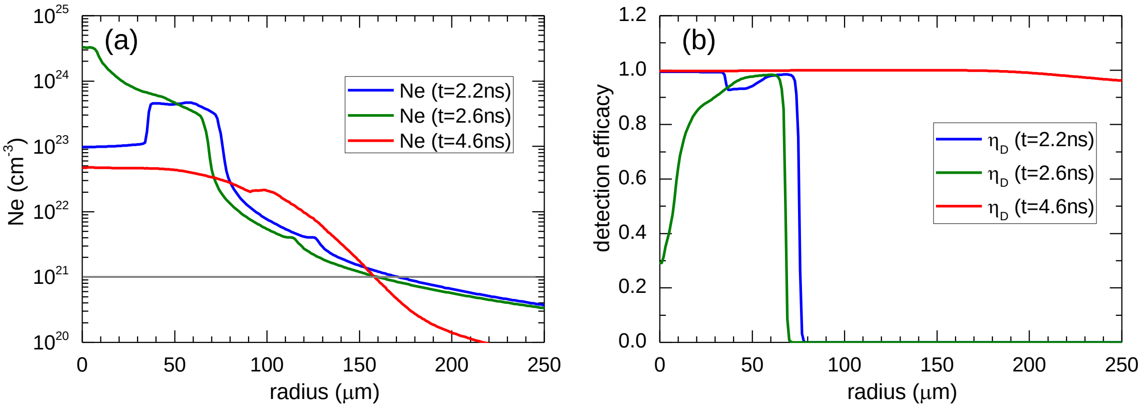

Supplementary Figure 1 | Simulated electron density and Kα imager detection efficiency. The radial distributions of (a) electron density and (b) Kα imager detection efficiency are obtained from FLASH simulations at different times: 2.2 ns (blue), 2.6 ns (green), and 4.6 ns (red). The critical density for the LFEX laser is marked by a horizontal line in (a).

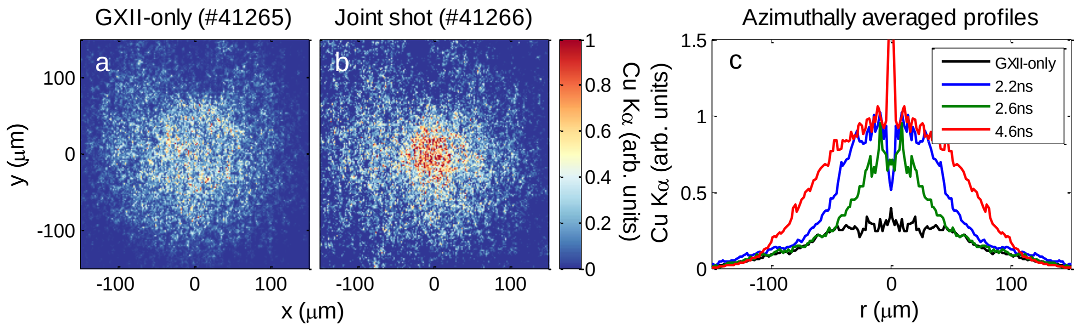

Supplementary Figure 2 | Experimentally measured Cu Kα images. These Cu Kα images are measured in (a) a GXII-only shot and (b) a joint shot, respectively. Summarized in (c) are azimuthally averaged profiles of the measured Cu Kα images from a GXII-only shot (black) and three joint shots with the LFEX laser injected at 2.2 ns (blue), 2.6 ns (green) and 4.6 ns (red), respectively.

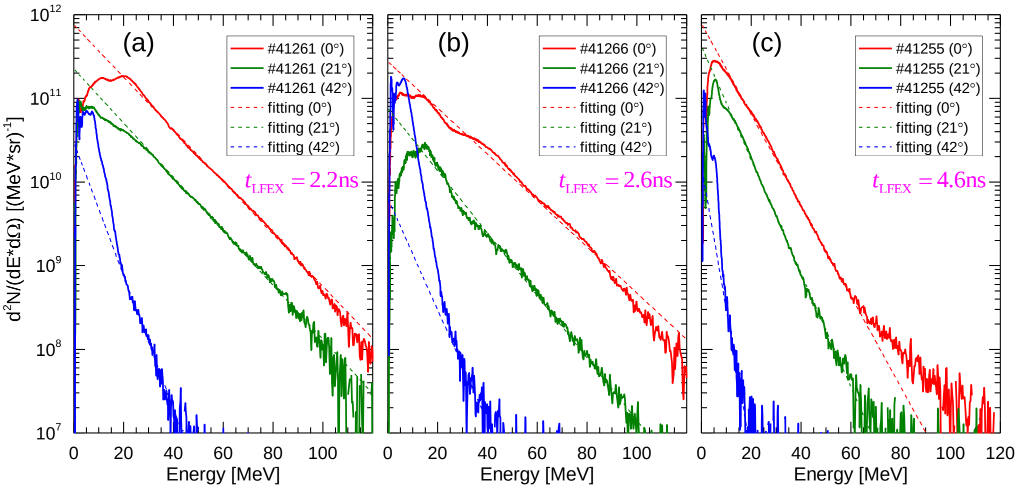

Supplementary Figure 3 | Experimentally measured energy spectra of fast electrons. These energy spectra of fast electrons are measured at directions of 0° (red solid), 21° (green solid) and 42° (blue solid) relative to the LFEX axis when the LFEX laser is injected at different times: (a) 2.2 ns, (b) 2.6 ns and (c) 4.6 ns. The dashed line is the exponential fitting of each spectrum. A clear 2-T exponential distribution is observed in the 42° spectra.

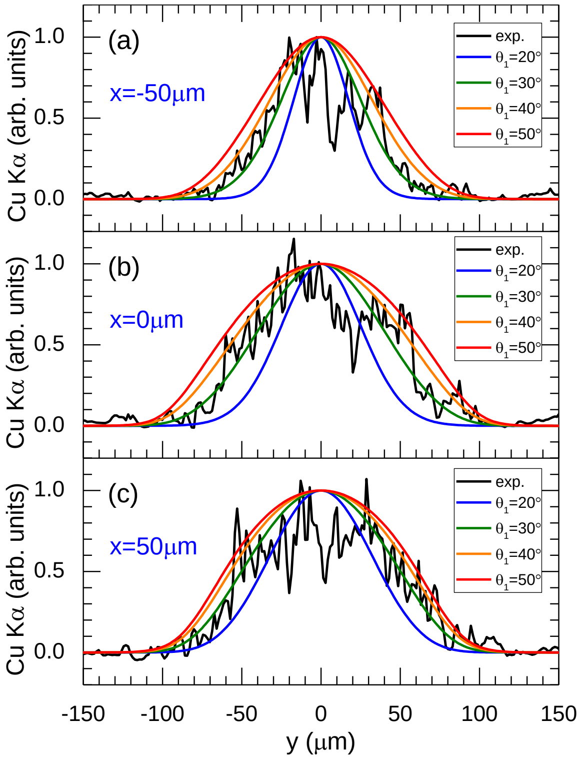

Supplementary Figure 4 | Transverse lineouts of a Cu Kα image. These normalized lineouts represent the transverse distributions of the Cu Kα image for $t_{\mathrm{LFEX}} = 4.6$ ns at (a) x=-50 μm, (b) x=0 μm, and (c) x=+50 μm, respectively. The black lines are from the experimental data (see **Figure 4c** in the main text); while the colored lines are from simulations with different divergence angles (θ_1) of the T_1-component fast electrons: 20° (blue), 30° (green), 40° (orange), and 50° (red).

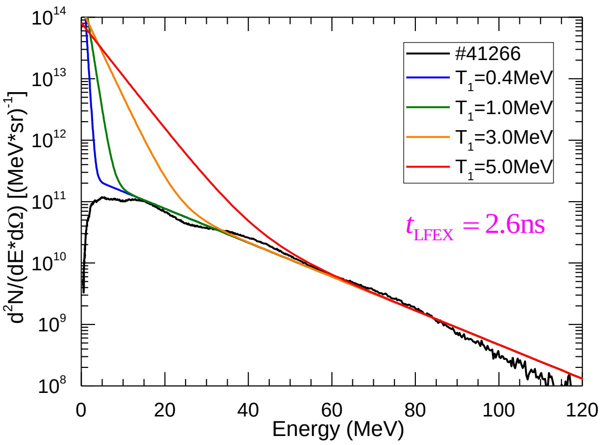

Supplementary Figure 5 | On-axis energy spectra of fast electrons. The colored lines are on-axis (0°) energy spectra of fast electrons used in simulations for the $t_{\mathrm{LFEX}} = 2.6$ ns case with different T_1: 0.4 MeV (blue), 1.0 MeV (green), 3.0 MeV (orange) and 5.0 MeV (red). The experimentally measured spectrum is plotted in black.

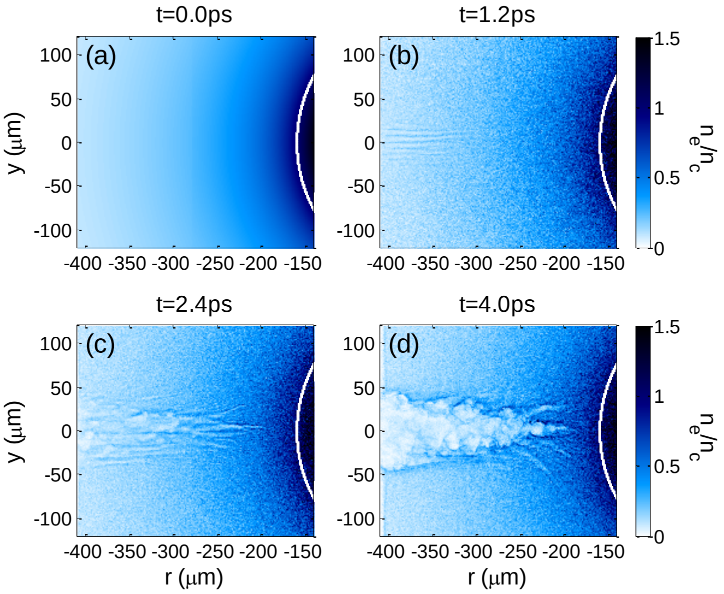

Supplementary Figure 6 | Particle-in-cell simulation results for the $t_{\mathrm{LFEX}} = 2.6$ ns case. Electron density profiles from the 2D PIC simulation for the $t_{\mathrm{LFEX}} = 2.6$ ns case are plotted at different times: (a) t=0.0 ps, (b) t=1.2ps ps, (c) t=2.4 ps, and (d) t=4.0 ps. The horizontal axis denotes the distance from the target center. The white contour represents the critical density (n_c) surface at t=0.0 ps.

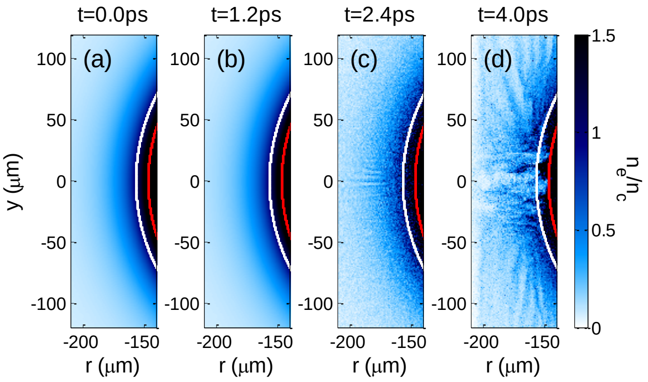

Supplementary Figure 7 | Particle-in-cell simulation results for the $t_{\mathrm{LFEX}} = 4.6$ ns case. Electron density profiles from the 2D PIC simulation for the $t_{\mathrm{LFEX}} = 4.6$ ns case are plotted at different times: (a) t=0.0 ps, (b) t=1.2ps ps, (c) t=2.4 ps, and (d) t=4.0 ps. The horizontal axis denotes the distance from the target center. The white and red contours represent the critical density (n_c) and the relativistic critical density (γn_c) surfaces at t=0.0 ps, respectively.

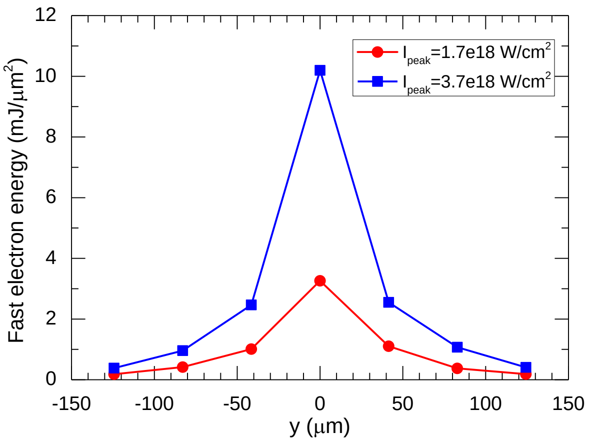

Supplementary Figure 8 | Transverse distributions of fast electron energies. These transverse distributions of the time-integrated fast electron energies are measured at the rear side of the simulation box for the $t_{\mathrm{LFEX}} = 4.6$ ns case with different peak intensities of the LFEX laser: 1.7×10^{18} W cm^{-2} (red dots) and 3.7×10^{18} W cm^{-2} (blue squares). These measurements only take into account the electrons with energy above 0.6 MeV.

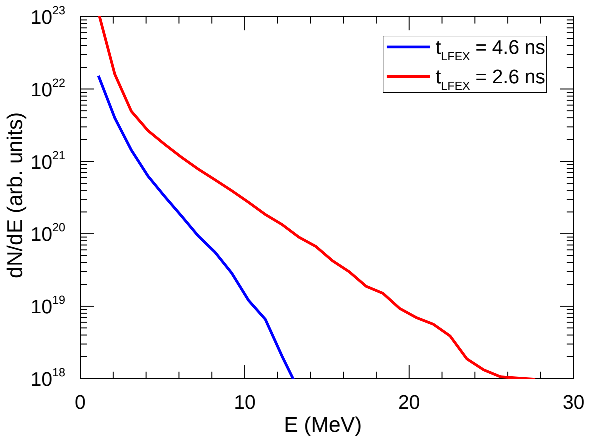

Supplementary Figure 9 | Energy spectra of fast electrons given by PIC simulations. The red and blue curves represent the results for the $t_{\mathrm{LFEX}} = 2.6$ ns and $t_{\mathrm{LFEX}} = 4.6$ ns cases, respectively. A clear feature of two-temperature is observed in these simulated spectra.

Supplementary Discussion

Radial distributions of plasma parameters from FLASH simulations

The simulated radial distributions of electron density and Kα imager detection efficiency at different times are displayed in **Supplementary Figure 1**. These results show that the critical density (10^{21} cm^{-3}) surfaces for the short pulse LFEX laser are respectively 170 μm (2.2 ns), 160 μm (2.6 ns), and 160 μm (4.6 ns) from the target center. At this critical density surface, the density gradient scalelengths at 2.2 ns and 2.6 ns are both ~60 μm, much larger than the ~15 μm at 4.6 ns. At 2.6 ns, the temperature at the target center is enhanced up to 200 eV (see **Figure 3b** in the main text); as a result, the Cu Kα line shifts and broadens, which greatly reduces the detection efficiency of the Kα imager as shown in **Supplementary Figure 1** (b). While at 2.2 ns and 4.6 ns, the core temperature is <70 eV (see **Figure 3b** in the main text), resulting in negligible effect on the detection efficiency. The dependence of the

detection efficiency on the plasma temperature used here is given by H. Sawada *et al.*[1] for a similar detector.

Cu Kα images measured in GXII-only and joint shots

Typical Cu Kα images measured in GXII-only and joint shots are shown in **Supplementary Figure 2** (a)-(b). The signal recorded in the GXII-only shot is attributed to the Cu Kα emission stimulated by GXII-produced suprathermal electrons as well as to the target self-emission in the energy range detectable by the Kα imager (8048±2.5 eV). When the LFEX laser is applied in the joint shot, clear enhancement in the core region caused by the fast electrons is observed, which is more evident in the azimuthally average profiles as shown in **Supplementary Figure 2** (c). The size of the Cu Kα emission attributable to the fast electrons changes with the LFEX injection time. But the emission in the outer region (e.g., r>50 μm for the 2.6ns case) always agrees with that in the GXII-only shot, indicating a good repeatability in the Cu Kα emission due to the GXII lasers. By subtracting the background measured in the GXII-only shot from the images measured in the joint shots, the fast electron induced Cu Kα images can be obtained.

Electron spectra measured by ESMs

The energy spectra of escaping fast electrons were measured by three absolutely calibrated ESMs, as shown in **Supplementary Figure 3**. By fitting these spectra with an exponential function, the on-axis (0°) slope temperatures and populations are obtained:

$T_2 = 13.9\ \mathrm{MeV}\ (2.2\ \mathrm{ns})$, $15.7\ \mathrm{MeV}\ (2.6\ \mathrm{ns})$, $8.0\ \mathrm{MeV}\ (4.6\ \mathrm{ns})$ and $N_2 = 1.1 \times 10^{13}\ \mathrm{sr}^{-1}\ (2.2\ \mathrm{ns})$, $4.3 \times 10^{12}\ \mathrm{sr}^{-1}\ (2.6\ \mathrm{ns})$, $6.3 \times 10^{12}\ \mathrm{sr}^{-1}\ (4.6\ \mathrm{ns})$, respectively. By fitting the populations from different directions with a Gaussian distribution, the divergence angles in FWHM are obtained: $\theta_2 = 33.2^\circ\ (2.2\ \mathrm{ns})$, $30.7^\circ\ (2.6\ \mathrm{ns})$, $33.0^\circ\ (4.6\ \mathrm{ns})$. The low temperature at 4.6 ns is caused by two factors: i) the low LFEX energy (191 J, compared to 396 J at 2.2 ns and 423 J at 2.6 ns), and ii) the short density scalelength (~15 μm, compared to ~60 μm at 2.2 ns and 2.6 ns, as shown in **Supplementary Figure 1** (a)).

If the fast electron beam characterized by these fitted parameters is used to calculate the stimulated Cu Kα emission, the intensity would be more than two orders of magnitude lower than the experimental data. It indicates that the fast electrons have another low-temperature (T_1) component, as implied by the spectra at 42° (blue lines). This T_1-component cannot be measured at 0° and 21°, probably because they are stopped by collisions or deflected by electric/magnetic fields when propagating through the high-density core. The 42° ESM measures the fast electrons transmitting through the coronal region (instead of through the core), thereby having a chance to observe the T_1-component. Since the on-axis T_1-component might be different from that at 42° and the former plays a dominant role in the Cu Kα emission and energy deposition, we set T_1 as a free parameter rather than use the one measured at 42°.

<u>Transverse lineouts of the Cu Kα image for $t_{\mathrm{LFEX}} = 4.6\ \mathrm{ns}$</u>

The large size of Cu Kα image measured at 4.6ns makes it possible to estimate the

divergence angle of the fast electron beam. This can be achieved by comparing the transverse lineouts of the Cu Kα image at different longitudinal locations between the experimental data and the simulation results, as shown in **Supplementary Figure 4**. The comparison indicates that the divergence angle of the fast electron beam at this LFEX injection time is $30^{\circ}{\sim}40^{\circ}$ in FWHM.

Parameters of fast electrons for $t_{\mathrm{LFEX}} = 2.6\ \mathrm{ns}$

Although the on-axis ESM is unable to measure the T_1-component of fast electrons, it can help us to estimate the upper limit of T_1. As shown in **Supplementary Figure 5**, the measured spectrum for $t_{\mathrm{LFEX}} = 2.6\ \mathrm{ns}$ (black) agrees well with an exponential distribution when the energy is above 20 MeV. It indicates that the contribution of T_1-component to the spectrum above 20 MeV is negligible. Two-temperature energy spectra of fast electrons with different T_1 are plotted in colored lines. These spectra are constrained by reproducing the experimentally measured Cu Kα image and Cu Kα photon number. As can be seen, only when $T_1 < 3\ \mathrm{MeV}$, the T_1-component contribution to the spectrum above 20 MeV is negligible. Therefore, in this case, an upper limit of 3 MeV for T_1 is inferred. Additionally, we choose the slope temperature given by the ponderomotive scaling[2] as the lower limit of T_1, which is ~0.5 MeV for the LFEX intensity in this case.

On the other hand, since the density scalelength in the coronal region at 2.6 ns is larger than that at 4.6 ns [see **Supplementary Figure 1** (a)], the divergence angle of fast electrons in the former case is expected to be larger than that in the later ($30^{\circ}{\sim}40^{\circ}$, see

Supplementary Figure 4)[3]. Therefore, a lower limit of 30° is chosen for the divergence angle, θ_1.

2D PIC simulation results for $t_{\mathrm{LFEX}} = 2.6$ ns and $t_{\mathrm{LFEX}} = 4.6$ ns

Two-dimensional PIC simulations were performed with the experimental LFEX parameters and the FLASH-simulated plasma conditions for the $t_{\mathrm{LFEX}} = 2.6$ ns and $t_{\mathrm{LFEX}} = 4.6$ ns cases. The LFEX laser pulse with a FWHM of 1.5ps was peaked at $t = 1.9$ ps and terminated at $t = 3.8$ ps. The electron density profiles at different times for these two cases are, respectively, shown in **Supplementary Figure 6** and **Supplementary Figure 7**. For the $t_{\mathrm{LFEX}} = 2.6$ ns (peak compression) case, where the density scalelength of the plasmas is very large (~60 μm), significant laser filamentation and channel bifurcation take place [see **Supplementary Figure 6** (b)-(c))], resulting in the plasma channel stopping in the underdense region when the LFEX laser terminates [see **Supplementary Figure 6** (d)]. It means the electron source has a large distance from the target center. In contrast, for the $t_{\mathrm{LFEX}} = 4.6$ ns case, where the density scalelength of the plasmas is very small (~15 μm), the plasma channel front is capable of arriving at the relativistic density surface [**Supplementary Figure 7** (d)] due to weak laser filamentation and channel bifurcation.

To explore whether the laser intensity plays an important role in the channel formation, another simulation for the case of $t_{\mathrm{LFEX}} = 4.6$ ns has also been performed with an enhanced laser intensity (3.7×10^{18} W cm^{-2}, same as that in the $t_{\mathrm{LFEX}} = 2.6$ ns case). The results are similar to those in **Supplementary Figure 7**, showing a weak dependence of channel

formation on the laser intensity. Additionally, the transverse distributions of the fast electrons for this $t_{\mathrm{LFEX}} = 4.6\ \mathrm{ns}$ case with different LFEX intensities are also similar to each other except for the absolute intensity, as shown in **Supplementary Figure 8**. It means that the transverse distribution of the fast electrons also depends weakly on the laser intensity. These results exclude the possibility that the low laser intensity in the $t_{\mathrm{LFEX}} = 4.6\ \mathrm{ns}$ case is responsible for the well collimated fast electrons. Therefore, it can be concluded that a well collimated fast electron beam can be produced by interacting a high intensity laser with a short density scalelength plasma. This conclusion is validated by the experimentally measured divergence angle ($30^{\circ}{\sim}40^{\circ}$ in FWHM) of the fast electrons for the $t_{\mathrm{LFEX}} = 4.6\ \mathrm{ns}$ case, as shown in **Supplementary Figure 4**.

The energy spectra of fast electrons for the $t_{\mathrm{LFEX}} = 2.6\ \mathrm{ns}$ and $t_{\mathrm{LFEX}} = 4.6\ \mathrm{ns}$ cases in PIC simulations are displayed in **Supplementary Figure 9**. It is worth noting that our PIC simulation is unable to angularly resolve the energy spectra of fast electrons. The results shown here are actually obtained by counting all the electrons escaping from the exiting boundary of the simulation box. Besides, the simulation is performed in 2D planar geometry, which is different from the 3D geometry in experiments. Therefore, one can not expect to compare the absolute quantities (e.g. population and slope temperature) between the simulation and the experiment. But the comparison of the relative tendency could be instructive. For instance, the simulated energy spectra display a clear two-temperature feature, which validates our introduction of a low-temperature (T_1) component in the fast electrons when analyzing the Cu Kα emission and the core heating. Additionally, the simulations show

a cooler temperature in the T_2-component in the $t_{\mathrm{LFEX}} = 4.6\,\mathrm{ns}$ case than that in the $t_{\mathrm{LFEX}} = 2.6\,\mathrm{ns}$ case, which is consistent with the experimental results as shown in **Supplementary Figure 3**.

According to these PIC simulations, the energy conversion efficiency from the LFEX laser to fast electrons (with energy above 0.6 MeV) for the $t_{\mathrm{LFEX}} = 2.6\,\mathrm{ns}$ case is 40.8%, much higher than that for the $t_{\mathrm{LFEX}} = 4.6\,\mathrm{ns}$ case (11.8%). Therefore, the laser-to-electron conversion efficiency seems not to be the reason for the low energy coupling efficiency measured in the experiment.

Supplementary References

1 Sawada, H. *et al.* Characterizing the energy distribution of laser-generated relativistic electrons in cone-wire targets. *Phys. Plasmas* **19**, 103108 (2012).

2 Wilks, S. C., Kruer, W. L., Tabak, M. & Langdon, A. B. Absorption of ultra-intense laser pulses. *Phys. Rev. Lett.* **69**, 1383-1386 (1992).

3 Peebles, J. *et al.* Investigation of laser pulse length and pre-plasma scale length impact on hot electron generation on OMEGA-EP. *New J. Phys.* **19**, 023008 (2017).
